# Subthreshold Electrical Noise Applied to the Plantar Foot Enhances Lower-Limb Cutaneous Reflex Generation

**DOI:** 10.3389/fnhum.2020.00351

**Published:** 2020-08-26

**Authors:** Tushar Sharma, Ryan M. Peters, Leah R. Bent

**Affiliations:** ^1^Department of Human Health and Nutritional Sciences, University of Guelph, Guelph, ON, Canada; ^2^Faculty of Kinesiology, University of Calgary, Calgary, AB, Canada

**Keywords:** stochastic resonance, cutaneous reflex, subthreshold noise, foot sole, lower limb

## Abstract

Reflex responses generated by cutaneous mechanoreceptors of the plantar foot are important for the maintenance of balance during postural tasks and gait. With aging, reflex generation, particularly from fast adapting type I receptors, is reduced, which likely contributes to impaired postural stability in this population. Therefore, improving reflex generation from these receptors may serve as a tool to improve balance performance. A mechanism to enhance reflexes may lie in the phenomenon of stochastic resonance, whereby the addition of certain intensities and frequencies of noise stimuli improves the performance of a system. This study was conducted to determine whether tactile noise stimuli could improve cutaneous reflex generation. In 12 healthy young adults, we evoked cutaneous reflex responses using a 0–50 Hz Gaussian noise vibration applied to the plantar heel. Concurrently, we applied one of six subthreshold intensities of electrical tactile noise to the plantar heel [0%, 20%, 40%, 60%, 80% or 100% (threshold)] and were able to analyze data from 0%, 20% and 40% trials. Across participants, it was found that the addition of a 20% perceptual threshold (PT) noise resulted in enhanced reflex responses when analyzed in both the time and frequency domains. These data provide evidence that cutaneous reflex generation can be enhanced *via* a stochastic resonance effect and that 20% PT is the optimal intensity of noise to do so. Therefore, the addition of noise stimuli may be a valuable clinical intervention to improve reflex responses associated with postural balance in populations with impairments.

## Introduction

Sensory information from the cutaneous mechanoreceptors of the foot sole is important for the successful maintenance of standing balance (Magnusson et al., 1990; Day and Cole, 2002) and gait (Yang and Stein, 1990; Zehr et al., 2014). While the specific nature of the information provided remains unclear, during natural postural sway and the stance phases of gait, these mechanoreceptors become activated and modulate muscle activity through cutaneous reflex loops (Nakajima et al., 2006; Zehr et al., 2014). Cutaneous reflexes evoked using electrical stimuli applied directly to the skin (Nakajima et al., 2006; Sayenko et al., 2009) or peripheral nerves housing cutaneous afferents (Aniss et al., 1992; Gibbs et al., 1995; Zehr et al., 2001a, 2014) elicit reflexive modulation of muscle activity in the lower limb with early (~50–70 ms), middle (~70–110 ms) and long (>110 ms) latencies. The early and middle latency responses are thought to represent spinal reflexes (Jenner and Stephens, 1982; Burke et al., 1991). The long-latency reflex may involve transcortical pathways (Jenner and Stephens, 1982; Burke et al., 1991). The middle latency reflex is of particular interest as it is thought to be most sensitive to modulation (Duysens and Tax, 1994; Zehr et al., 2001b). For example, the middle latency reflex exhibits a location dependence, such that activation of mechanoreceptors in different foot regions results in different patterns of muscle activity modulation (Sonnenborg et al., 2000; Nakajima et al., 2006; Sayenko et al., 2009). In particular, when an electrical cutaneous stimulation is applied to the heel region, an excitatory response occurs in the soleus (a foot plantar flexor muscle) and an inhibitory response occurs in the soleus’ antagonist, the tibialis anterior (a foot dorsiflexor). When the stimulation is moved anteriorly to the metatarsal region, a reversal of these reflexes occurs such that the soleus response becomes inhibitory and an excitatory reflex occurs in the tibialis anterior (Sonnenborg et al., 2000; Nakajima et al., 2006; Sayenko et al., 2009). This pattern of activation may function to remove the foot from obstacles that may contact the plantar foot during postural balance or gait (Nakajima et al., 2006). In gait, the amplitude of cutaneous reflexes in a muscle also depends on the phase of gait during which they are evoked (Duysens et al., 1990; Yang and Stein, 1990; De Serres et al., 1995; Zehr et al., 1997, 2014). Activation of cutaneous afferents through stimulation of the posterior tibial nerve produces responses that cause foot dorsiflexion during the late stance phase but foot plantarflexion during the late swing phase (Zehr et al., 1997). Similar patterns of activation occur following electrical stimuli applied to the heel (Zehr et al., 2014). This phase-dependency of cutaneous reflexes during gait is thought to be driven by central pattern generator activity (Klarner and Zehr, 2018). While all four types of cutaneous mechanoreceptors found in the glabrous skin of the foot can generate cutaneous reflex responses (Fallon et al., 2005; Bent and Lowrey, 2013), fast adapting type-I (FAI) receptors are believed to be particularly important for the maintenance of balance. Previous work has shown that FAI mechanoreceptors are the most densely populated type of mechanoreceptor across the foot sole (Kennedy and Inglis, 2002; Strzalkowski et al., 2018), and are the most synaptically-coupled mechanoreceptor to muscles of the lower (Fallon et al., 2005) and upper limb (Bent and Lowrey, 2013). However, in healthy adult aging, morphological and physiological changes occur to cutaneous mechanoreceptors. For example, FAI receptors are less densely populated (Bolton et al., 1966) and have reduced cross-sectional areas (Iwasaki et al., 2003). Such changes impair the sensory function of these receptors and agree with age-related reductions in the sensitivity of foot sole receptors that have previously been reported (Kenshalo, 1986; Wells et al., 2003; Peters et al., 2016). Impaired sensory input may also explain age-related reductions in FAI initiated cutaneous reflex responses in the lower limb that have been recently reported (Peters et al., 2016). By applying 30 Hz vibratory stimuli, which have been shown to preferentially activate FAI receptors in the foot (Strzalkowski et al., 2017), Peters et al. (2016) showed that in older adults, the ability of FAI mechanoreceptors to generate reflex responses was significantly impaired. This impaired ability to generate responses may contribute to the increased risk of falls that has been described in the older adult population (Peters et al., 2016). Therefore, enhancing the ability of these cutaneous mechanoreceptors to generate reflex responses may be a viable method of reducing the risk of falls in older adults.

Stochastic resonance is a phenomenon whereby the addition of subthreshold noise to a system improves its performance (Collins et al., 1995, 1996; Richardson et al., 1998; Wells et al., 2005). Only certain intensities of noise aid the system’s performance; in theory there exists an optimal intensity of noise that results in the greatest performance enhancements. Intensities below or above the optimal level either provide enhancement to a lesser degree, no enhancement at all, or may even make the signal degrade. Further, the frequency content of the noise stimulus has been shown to influence the strength of noise-related performance enhancements (Trenado et al., 2014b). Therefore, the stochastic resonance phenomenon is noise-intensity and frequency-content dependent. In the cutaneous somatosensory system, imperceptible tactile vibration and electrical noise stimuli applied to the skin of the hand (Collins et al., 1996, 1997; Richardson et al., 1998) and feet (Dhruv et al., 2002; Wells et al., 2005) increases the sensitivity of mechanoreceptors to tactile stimuli. Such improvements in perceptual threshold have been observed in young adults (Collins et al., 1996, 1997; Richardson et al., 1998; Wells et al., 2005), healthy older adults (Dhruv et al., 2002; Liu et al., 2002; Wells et al., 2005), and populations suffering from stroke or diabetes-related sensory deficits (Liu et al., 2002). Tactile noise has also been shown to improve functional outcomes such as postural sway in similar populations (Priplata et al., 2002, 2003, 2006; Galica et al., 2009; Lipsitz et al., 2015). Currently, however, it is unknown whether functional enhancements are due to improved cutaneous reflex generation. Thus, this study aimed to determine whether subthreshold tactile stimuli could be used to enhance cutaneous reflex responses in muscles of the lower limb. Additionally, we aimed to determine whether an ideal noise intensity exists to optimally enhance cutaneous reflex responses. We hypothesized that tactile noise would enhance the ability of plantar surface cutaneous mechanoreceptors to generate reflex responses. This would be observed as a facilitation of soleus reflex responses and reductions in tibialis anterior responses, based on location dependency (Nakajima et al., 2006). Additionally, we hypothesized that there would be a specific intensity of noise that would optimally enhance cutaneous reflex generation. At this intensity, reflex magnitudes were expected to be at their highest in the soleus while being at their lowest in the tibialis anterior. Preliminary findings from this work have been published in abstract form (Sharma et al., 2019).

## Materials and Methods

### Participants

Twelve young adults (nine females; mean age ± SD: 22.5 ± 2.0 years) participated in this study. Participants were screened to ensure the absence of any neuromuscular disorders before participation. All participants provided informed, written consent, and all procedures were approved by the University of Guelph Research Ethics Board.

### Experimental Setup

**Figure 1:**
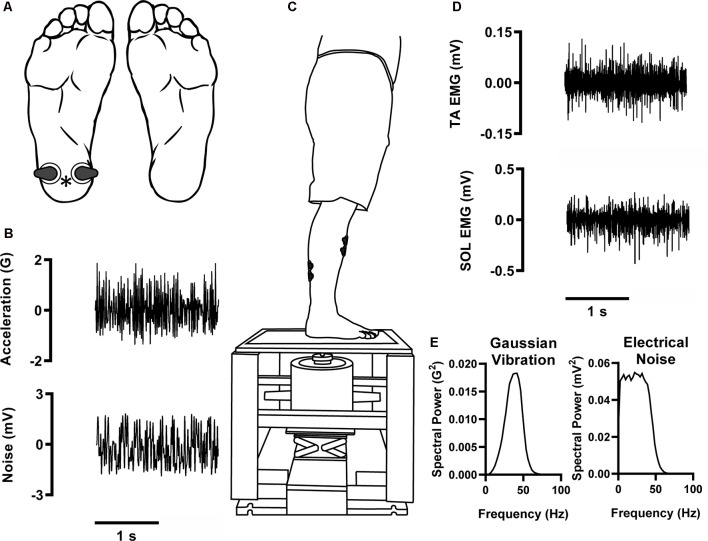
Experimental setup. Participants stood on a custom-built platform with a slight anterior lean **(C)**. Two-second data excerpts of input stimuli from a 120-s trial. Gaussian vibration stimuli [**B**, intensity:10× perceptual threshold (PT); **E**, spectral power] were applied to the plantar heel of the right foot (indicated by *) to evoke cutaneous reflexes **(A)**. Electrical noise stimuli were concurrently applied at intensities between 0% and 100% PT (**A**, location; **B**, two-second waveform excerpt; **E**, spectral power). **(D)** Two-second data excerpts of recorded electromyography (EMG) signals from a 120-s trial. EMG was recorded from the soleus (SOL) and tibialis anterior (TA).

During experimentation, participants stood on a custom-made platform that supported them over a vibration motor (type 4808 Magnetic Vibration Exciter and power amplifier type 2719, Brüel and Kjær Sound and Vibration Measurement A/S, Nærum, Denmark). Attached to this motor was an accelerometer (type 4507, Brüel and Kjær Sound and Vibration Measurement A/S, Nærum, Denmark) and a metal probe (~5 mm diameter) that passed through a hole in the platform to contact the participant’s heel and vibrate perpendicular to the foot (Figure 1). Probe acceleration was sampled at 5,000 Hz. To ensure the probe activated a similar population of mechanoreceptors between trials an outline of a box (2 cm × 2 cm) was made on the foot. Before every trial, an experimenter ensured that the probe was in contact with the skin within this outline.

Surface electromyography (EMG; AMT-8 system, Bortec Biomedical Limited, Calgary, AB, Canada) was measured from the soleus (SOL) and tibialis anterior (TA) by affixing two Ag/AgCl electrodes over each muscle belly (Figure 1C). EMG data were bandpass filtered (10–1,000 Hz) and sampled at 5,000 Hz.

### Stimulation and Threshold Testing

Cutaneous reflexes were evoked by applying a continuous Gaussian vibration stimulus consisting of random frequencies between 0 and 50 Hz to the heel (Figure 1B; Type 4808 Magnetic Vibration Exciter, Brüel and Kjær Sound and Vibration Measurement A/S, Nærum, Denmark). An aperiodic vibration was chosen as it better represents the nature of stimuli cutaneous mechanoreceptors respond to during activities of daily living (Collins et al., 1997). This frequency band was chosen to preferentially activate FAI mechanoreceptors (Strzalkowski et al., 2017). To determine the perceptual threshold (PT), we employed a modified method of limits; briefly, the intensity of a 0–50 Hz Gaussian vibration was increased until the participant could faintly feel the stimulus on the region of interest (heel). We then lowered the intensity until the stimulus was no longer detectable by the participant. This was repeated three to four times and the lowest intensity that consistently caused a percept was defined as PT [mean ± SD; represented as acceleration relative to gravity (G)] = 0.36 ± 0.34 G. For testing we used a vibration amplitude that was 10× PT to ensure a high level of cutaneous activation. It should be noted that at 10× PT, our motor produced a faint sound that could be heard by the participant. However, a vibration intensity of 10× PT was used in every trial. Therefore, the intensity of this sound was the same in every trial and likely did not influence reflex amplitudes.

To enhance the generated cutaneous reflexes, we concurrently applied electrical band-pass filtered (0.01–50 Hz) white noise stimuli to the heel (Figure 1B; A395 Linear Stimulus Isolator, World Precision Instruments, Sarasota, FL, USA). Electrical PTs for all participants were found to be within a stimulus peak-to-peak (PTP) amplitude of 20 mA. The intensity of the electrical noise stimulus varied between trials and was set to 0% (no noise, control), 20%, 40%, 60%, 80%, or 100% of PT. PT for this electrical stimulus was measured separately from the vibration but followed the same procedures. PT was established when the electrical stimulation was felt to just perceptibly radiate over the plantar heel area.

### Experimental Procedures

During each trial, participants stood and were asked to maintain a slight anterior lean, to introduce a level of background activity corresponding to 20% of soleus root mean squared maximal EMG, which was determined by performing a maximal voluntary plantarflexion contraction at the onset of the trial. During standing, the previously described Gaussian noise vibration was applied to the heel by the vibrating probe at 10 times the perceptual threshold (10× PT). Concurrently, the continuous, electrical noise stimulus was applied to the plantar skin of the heel through electrodes placed on either side of the probe. The intensity of the electrical noise stimulus varied between trials and was set randomly to one of 0% (no noise), 20%, 40%, 60%, 80% or 100% of PT. Participants were not informed of the intensity of noise applied in any trials. Two–120-s trials were performed with each electrical noise intensity in a randomized order for a total of 12 trials (two trials × six noise intensities). Seated breaks were taken between each trial (every 2 min) to prevent muscular fatigue between trials. All trials were conducted in a single testing session.

### Data Analysis

All analyses were performed in MATLAB software (MathWorks, Natick, MA, USA). Data were initially visually inspected to ensure there were no stimulus artifacts that contaminated any recorded signals. If stimulus artifacts were present in a participant’s EMG data, EMG data from all noise conditions were removed from the contaminated muscle’s analysis. When generating cumulant density and coherence functions for conditions with contaminated EMG signals, the presence of stimulus artifact was pronounced. Since the electrical noise and the vibration stimuli are applied concurrently, a large spike in the cumulant density is observed at 0 ms. Additionally, since both stimuli contain frequencies between 0–50 Hz, strong coherence is observed across the whole 0–50 Hz band. Of the 12 participants, tibialis anterior data from two participants were excluded due to excessive noise in the signal which prevented analysis. Additionally, during the higher electrical noise intensity conditions (60–100% PT) stimulus artifact contaminated both SOL and TA EMG data for all participants. For this reason, only 0%, 20%, and 40% PT trials were compared.

For analysis, we concatenated probe acceleration, SOL EMG, and TA EMG from both trials at each noise intensity for each participant. Concatenated probe acceleration data were low-pass, zero-lag filtered at 50 Hz (4th order Butterworth). Concatenated SOL EMG and TA EMG data were full-wave rectified. A modified version (based on Dakin et al., 2010) of NeuroSpec code (version 2.0; www.neurospec.org) was used to assess the spectral characteristics of the evoked cutaneous reflex responses in the frequency and time domain for each participant (292 disjointed segments, segment length = 0.8192 s, frequency resolution = 1.2207 Hz). The specific mathematical processes involved in these analyses are outlined in Halliday et al. (1995), but are briefly described here. In the frequency domain, coherence functions were generated. The coherence function provides a measure of linear association between an input (i.e., vibratory probe acceleration) and an output (i.e., EMG) signal at each frequency assessed. As a measure of association, coherence values are bounded between 0–1. For each coherence estimate, 95% confidence limits were generated by the Neurospec2.0 code (described in Halliday et al., 1995). Values exceeding this limit were considered significantly different from 0 (0 represents no coherence), suggesting significant coherence at that specific frequency (Halliday et al., 1995). From each coherence function, we measured the peak coherence between 28–32 Hz which encompasses frequencies that preferentially target FAI receptors (Strzalkowski et al., 2017). Further, these were frequencies with statistically significant coherence estimates based on the pooled analyses (described further in this section). In the time domain, cumulant density functions were generated. Cumulant density functions provide a measure of linear dependence between an input and output signal in the time domain. Responses were considered significant if the cumulant density function exceeded the 95% confidence interval calculated by the Neurospec2.0 code based on the inherent variability of the cumulant density function (Halliday et al., 1995). From individual cumulant density functions, PTP amplitudes of responses occurring between 70–110 ms were identified as the cutaneous reflex response (the time frame of a cutaneous reflex response in the lower limb). If a clear response was not generated in a condition, latencies of PTP amplitudes from the participant’s other conditions were used to determine the time frame over which to include data for the analysis. Measured PTP amplitudes in the 20% and 40% noise conditions were normalized to PTP amplitudes obtained in the control (0%) condition. To statistically compare mean PTP amplitudes and peak coherences across noise intensities, one-way repeated measures ANOVAs were performed in IBM SPSS (v26, IBM, Armonk, NY, USA). If the assumption of equal sphericity between conditions was violated (as assessed by Mauchly’s Test of Sphericity), a Greenhouse–Geisser correction was applied. If statistical significance was observed from the ANOVA, *post hoc* tests with a Bonferroni correction were performed.

We also performed a pooled analysis where probe acceleration, SOL EMG, and TA EMG data from all participants were concatenated at each noise intensity. Therefore, a total of 24 trials were concatenated for SOL analysis (12 participants × 2 trials; 3,515 disjointed segments, 0.8192-s segment length) and 20 trials for TA analysis (10 participants × 2 trials; 2,929 disjointed segments, 0.8192-s segment length) at each noise intensity. Pooled coherence at each frequency (0–50 Hz, 1.2207 Hz resolution) was statistically compared between noise intensity conditions in a pairwise manner by performing a difference of coherence (DoC) test. The DoC test is a statistical tool that assesses differences between coherence estimates at each frequency based on the Chi-squared distribution (Amjad et al., 1997). We were particularly interested in differences occurring within the 25–35 Hz bandwidth. Any differences exceeding 95% confidence limits at any frequencies were interpreted as being significantly different coherence estimates (Amjad et al., 1997). Pooled cumulant density functions were also generated and the PTP amplitude of the function was calculated between 70–110 ms (the time frame of a middle-latency cutaneous reflex response in the lower limb).

## Results

### Cumulant Density

**Figure 2:**
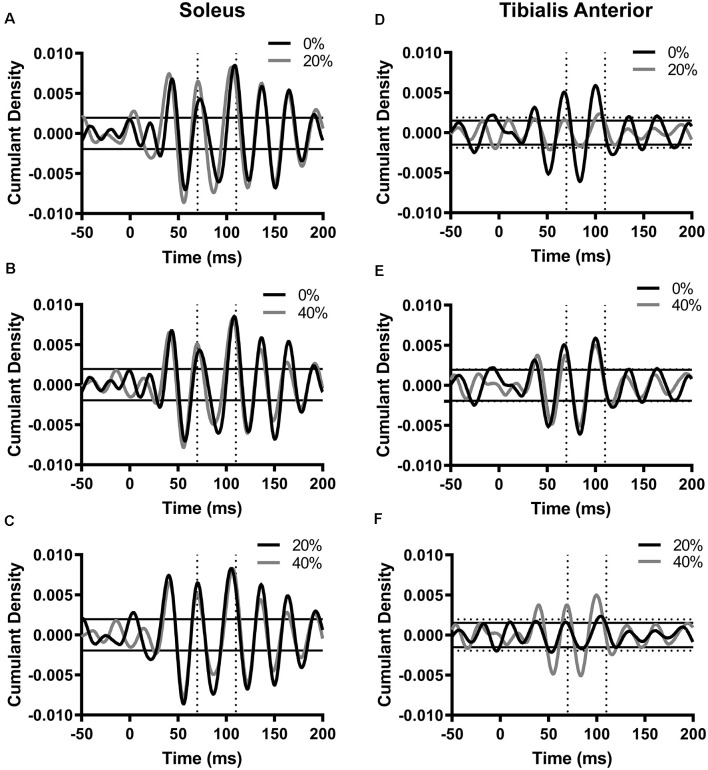
Cumulant density functions generated between probe acceleration and soleus EMG **(A–C)** or tibialis anterior EMG **(D–F)**. Horizontal lines represent 95% confidence interval bounds for the 0% PT condition. In figures **(D–F)**, solid horizontal lines represent confidence intervals for the cumulant density function in the 20% noise condition **(D,F)** or 40% noise condition **(E)**. Dotted horizontal lines represent confidence intervals for the cumulant density function in the 0% noise condition **(D)** or 40% noise condition **(E,F)**. For all cumulant densities, peak-to-peak (PTP) amplitudes for each function were calculated between 70–110 ms (indicated by the vertical broken lines). The number of segments: SOL, 3,515 disjointed segments; TA, 2,929 disjointed segments. Segment length = 0.8192 ms, time resolution: 0.02 ms.

To assess reflex gain, we measured PTP amplitude of the pooled cumulant density function within 70–110 ms and compared it across each noise intensity (Figure 2). With pooled data, significant reflex responses were generated in all three noise conditions in each muscle within this time window (*p* < 0.05). In soleus, PTP amplitude was 0.0146 at 0% (latency = 84 ms). When 20% PT noise was added, PTP amplitude increased to 0.0157 (7.67% increase from 0% PT, latency = 79.4 ms). At 40% PT, PTP amplitude decreased to 0.01319 (9.47% decrease from 0% PT, latency = 79.8 ms). In tibialis anterior, at 0% PT, PTP amplitude was 0.0120 (latency = 77.4 ms). The application of 20% PT noise resulted in a decrease in PTP to 0.00407 (66.0% decrease from 0% PT, latency = 81.4 ms). At 40% PT, PTP amplitude decreased to 0.0101 (15.9% decrease from 0% PT, latency = 77.8 ms).

Finally, to get a sense of individual data, we also measured PTP of responses in individual participants at each noise intensity in the 70–110 ms window (Figure 4). Mean soleus PTP amplitude was 13.7% larger in the 20% noise condition and 16.1% smaller in the 40% PT noise condition compared to 0% PT (Figure 4A). Mean tibialis anterior PTP amplitude was 1.0% and 18.9% greater in the 20% and 40% conditions respectively (Figure 4C). However, these differences were not statistically significant (SOL: *F*_(1.324,14.559)_ = 3.334, *p* = 0.08; TA: *F*_(2,18)_ = 0.273, *p* = 0.76).

**Figure 3:**
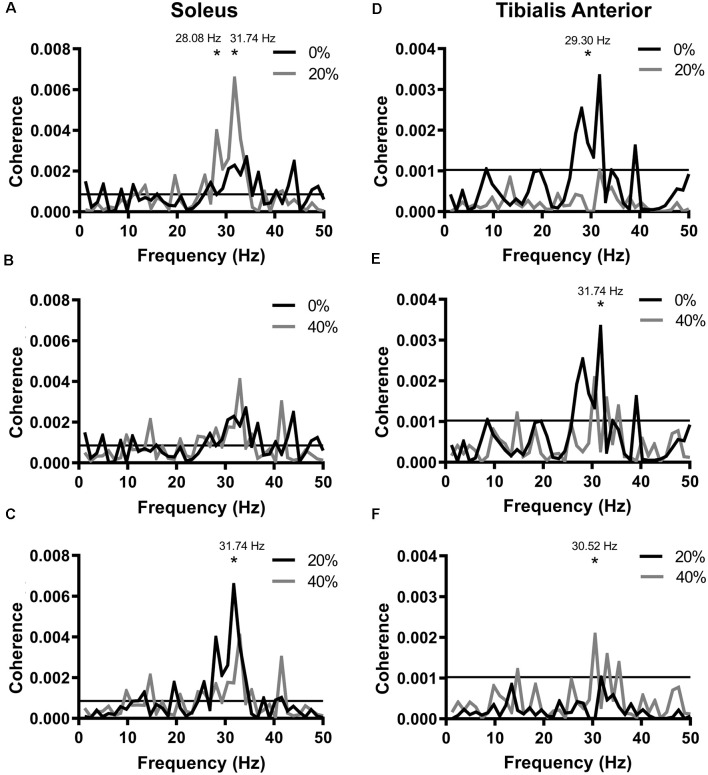
Coherence functions generated between probe acceleration and soleus EMG **(A–C)** or tibialis anterior EMG **(D–F)**. Horizontal lines represent the 95% confidence limit bounds. Frequency resolution: 1.2207 Hz. *Indicates significant differences between the two plotted coherence functions at the frequency indicated. The difference in coherence frequency resolution: 1.2207 Hz.

**Figure 4:**
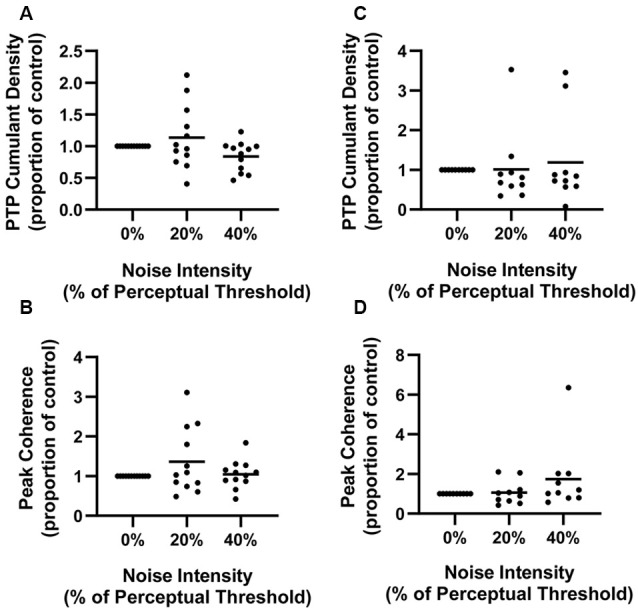
Normalized soleus cumulant density PTP amplitude **(A)** and peak soleus coherence between 28–32 Hz **(B)** and normalized tibialis anterior cumulant density PTP amplitude **(C)** and peak coherence between 28–32 Hz **(D)** at each noise intensity. Dots represent individual participant data at each noise (soleus: *n* = 12, tibialis anterior: *n* = 10) and the horizontal line represents the overall mean.

### Coherence

We also generated pooled coherence functions at each noise intensity and compared coherence across the 25–35 Hz frequency range (Figure 3). Significant coherence was observed within this frequency range in all conditions (0%, 20%, 40%). In SOL, peak pooled coherences were 0.00269 (observed at 34.18 Hz) at 0% PT, 0.00656 (31.74 Hz) at 20% PT and 0.00174 (31.74 Hz) at 40% PT. At 28.08 Hz, coherence in the 20% PT condition was significantly greater than at 0% PT (Figure 3A; $\chi_{\left(1\right)}^{2}$ = 4.045, *p* = 0.0443). At 31.74 Hz, pooled coherence at 20% PT was significantly greater than both the 0% PT (Figure 3A; $\chi_{\left(1\right)}^{2}$ = 3.911, *p* = 0.0480) and 40% PT conditions (Figure 3C; $\chi_{\left(1\right)}^{2}$ = 5.469, *p* = 0.0194). In TA, peak pooled coherences were 0.00333 (31.74 Hz) at 0% PT, 0.000595 (34.18 Hz) at 20% PT and 0.00102 (31.74 Hz) at 40% PT. At 29.30 Hz, pooled coherence in the 20% PT condition were significantly lower than in 0% PT (Figure 3D; $\chi_{\left(1\right)}^{2}$ = 4.160, *p* = 0.0414). Twenty percentage PT pooled coherence was less than 40% PT coherence at 30.52 Hz (Figure 3F; $\chi_{\left(1\right)}^{2}$ = 5.190, *p* = 0.0227). Pooled coherence in the 40% PT condition was significantly lower at 31.74 Hz than in the 0% PT condition (Figure 3E; $\chi_{\left(1\right)}^{2}$ = 4.898, *p* = 0.0269).

Means of individual peak coherences between 28–32 Hz were also measured (Figure 4). This frequency band was chosen as the DoC revealed statistically significant pooled coherence estimates between 28–32 Hz. In soleus, the mean of peak coherences was 36.3% and 4.4% greater in the 20% and 40% conditions respectively, when compared to no noise (Figure 4B). In tibialis anterior, the means of peak coherences were 6.5% and 73.8% greater than that at 0%, for the 20% and 40% noise conditions respectively. Contrary to the pooled analysis, differences in the individual coherence data were not statistically different (SOL: *F*_(1.173,12.907)_ = 1.941, *p* = 0.17; TA: *F*_(1.125,10.125)_ = 2.008, *p* = 0.19).

## Discussion

This study was conducted to determine whether the addition of subthreshold electrical tactile noise could enhance cutaneous reflex generation in the lower limb. We saw that the addition of electrical tactile noise at an intensity of 20% PT increased the magnitude of pooled cutaneous reflex responses and significantly increased pooled coherence at ~30 Hz in SOL. In contrast, TA saw a reduction in pooled cutaneous reflex magnitudes and significant reductions in pooled coherence at ~30 Hz. While significant reductions in pooled TA coherence continued to be observed in the 40% PT condition, a corresponding increase in pooled SOL coherence and pooled cumulant density PTP amplitude were not observed. Overall, these results may suggest that tactile electrical noise on the plantar surface of the foot can enhance naturally occurring cutaneous reflex responses in the plantar and dorsiflexor muscles and that the optimal intensity is 20% PT.

### Afferent Contribution to the Reflex Response

The vibration input applied to the skin to evoke cutaneous reflexes was a Gaussian noise stimulus with a bandwidth of 0–50 Hz. As such, the power across this frequency band is normally distributed with peak power occurring around 30 Hz. Of the four cutaneous mechanoreceptor types in the foot, vibration stimuli around this frequency preferentially target FAI receptors (Kennedy and Inglis, 2002; Strzalkowski et al., 2017). In our data, peak coherences between signals were observed around 30 Hz in all conditions. This likely suggests that the observed responses were generated primarily by FAI receptors. However, the 0–50 Hz bandwidth also contains frequencies shown to target slow-adapting type I (SAI) and type II (SAII) mechanoreceptors which preferentially respond to frequencies <20 Hz (Strzalkowski et al., 2017). Despite this, minimal coherence was observed between 0–20 Hz, suggesting minimal contributions from SA receptors to the observed reflex responses. This is likely a result of weaker power below 20 Hz in the vibration input. Some care must be taken when interpreting cutaneous contributions simply by frequency alone, as we have shown in our past work (Strzalkowski et al., 2017) that amplitude can also affect the ability of individual afferents to fire, particularly at the high amplitudes used here. However, even at high amplitudes (>2 mm), we showed that SA afferents do not contribute appreciably to frequencies above 10 Hz (Strzalkowski et al., 2017). Additionally, of the foot sole mechanoreceptor types, SAI and SAII receptors show the weakest ability to modulate the activity of lower limb motor units (Fallon et al., 2005). Finally, while fast-adapting type II receptors do respond to frequencies between 0–50 Hz, they likely did not contribute appreciably to the observed reflex responses due to relatively lower total receptor number (Strzalkowski et al., 2018) and weaker reflex coupling with lower limb muscles (Fallon et al., 2005). Therefore, we believe that the responses we observed were generated primarily by FAI receptors.

### Optimal Noise Intensity Is 20% PT

Based on the limited intensities of noise analyzed, we observed that electrical noise intensities around 20% PT are ideal to enhance cutaneous reflex generation. Due to stimulus artifact from the electrical noise stimulus contaminating much of the recorded EMG signals in the higher noise intensity conditions, only the 0%, 20% and 40% PT noise conditions were analyzed and compared. The cumulant density plot for these conditions did not contain a large spike at 0 ms and strong coherence was not present across the whole 0–50 Hz band. Therefore, we can be confident that the responses observed at 0%, 20%, and 40% are not due to artifact contamination and are a result of physiological processes.

While we cannot analyze the data at these higher intensities, we do not believe that a more ideal intensity exists above 40% PT. The typical stochastic resonance phenomenon involves the gradual enhancement of a system while increasing noise intensity up to an optimal intensity (McDonnell and Abbott, 2009). Increases in noise intensity beyond this intensity would cause a detrimental effect on system performance (McDonnell and Abbott, 2009). In our study, increasing noise intensity from 20% to 40% resulted in the reduction of the reflex response in SOL, closer to the no noise control condition. Therefore, increasing noise intensity beyond 40% would likely not facilitate reflex generation. Clearly, it is not possible to confirm a lack of enhancement in these higher noise conditions without including these intensities in our data analysis. However, we suggest that 20% of PT is optimal to enhance cutaneous reflex generation based on our current, potentially limited, data.

In previous studies that have explored the stochastic resonance effect in cutaneous mechanoreceptors, the optimal intensity of tactile noise is variable. In the foot, Dhruv et al. (2002) applied electrical noise to the metatarsal region of the plantar foot at intensities of 0%, 20%, 40%, 60%, and 80% of PT in healthy, older adults. When data from these noise trials were pooled and collectively compared to no noise, monofilament thresholds were lower in most participants. In their study, while enhancements were reported, no statistical comparisons were performed between these intensities and therefore no optimal intensity was established. Interestingly, the increased sensitivity is likely related to the improved sensory function of FAI receptors as these receptors have a strong correlation in their afferent firing in response to monofilament indentations of the skin (Strzalkowski et al., 2015).

Using vibrational noise, Wells et al. (2005) showed that in a healthy, young population, 33% PT noise is the optimal intensity of vibrational noise to enhance the detection of a subthreshold (0.9× PT) sinusoidal 25 Hz vibration (targeting FAI). However, 33% PT noise was the lowest non-zero intensity of noise that was tested. Interestingly, when the intensity of the sinusoid to be detected was decreased to 0.8× PT, the optimal noise intensity required to enhance the detection of the sinusoid increased to 50% PT. In this study by Wells, a subthreshold sinusoid signal was shown to be made detectable and the degree to which this signal is subthreshold affects the optimal intensity of noise. In our study, reflex responses are generated without the addition of noise, and therefore would likely require a lower intensity of noise to enhance such reflexes and may explain why the optimal noise intensity was a relatively lower intensity of noise.

Tactile noise has also been shown to improve measures of postural sway. However, these studies tend to apply relatively higher intensities of noise. For example, Priplata et al., 2002, 2003, 2006 showed improved postural balance with the addition of noise at an intensity of 90% PT. This intensity has also been shown to improve gait measures (Galica et al., 2009; Miranda et al., 2016). Lipsitz et al. (2015) applied tactile noise to the foot at intensities of 0%, 70%, and 85% PT and found that both 70% and 85% PT enhanced gait measures similarly. As these studies do not apply other intensities of noise, it is unknown whether a more optimal intensity exists below 70% PT.

### Functional Response

The pattern of activation that was observed following vibration at the heel, soleus excitation, and tibialis anterior inhibition, is consistent with other studies that have evoked cutaneous reflexes at the heel (Sonnenborg et al., 2000; Nakajima et al., 2006; Sayenko et al., 2009). This provides further support that cutaneous mechanoreceptors have excitatory and inhibitory input onto motorneuron pools innervating muscles of the lower limb. This pattern of activation is thought to be functionally significant for the initiation of stance during the step cycle. During heel contact, soleus excitation and tibialis anterior inhibition may allow the stance limb to appropriately maintain balance during the stance phase (Zehr et al., 1997, 2014). Soleus excitation and tibialis anterior inhibition may also allow for recovery following disturbances during gait. For example, if the heel comes in contact with an obstacle, activation of the soleus and inhibition of the tibialis anterior would together act to cause plantarflexion of the foot in an attempt to lift the foot away from the obstacle and allowing for the maintenance of balance during gait. Gait was not examined in the current experiment and so it is important to acknowledge that the true functional application of SR to gait characteristics requires further study.

In our study, the participants were young and healthy, and we were able to evoke reflex responses without the addition of noise. However, when tactile noise was added, the reflex generation was enhanced. The underlying mechanism is proposed to relate to the number of FAI mechanoreceptors involved in the generation of the response. During daily activities, mechanical input to the foot will activate mechanically-sensitive gates that will release neurotransmitters which open channels that will generate an electrical signal in the sensory nerve ending of the afferent nerve (Hao et al., 2015). Since electrical cutaneous stimuli do not include a mechanical component, such stimuli may directly target downstream voltage-gated channels (KCNQ, Nav1.9) within the sensory nerve ending that will facilitate the generation of action potentials (Hao et al., 2015). Since our electrical stimuli were applied below the perceptual threshold, these stimuli are proposed to elevate the resting membrane potential *via* these voltage-gated channels that are known to alter the gain of the afferent bringing these afferents closer to the voltage threshold required for action potential generation. Therefore, mechanoreceptors that would normally not be activated in response to the vibration input now can be activated due to this elevated and synchronized potential. Therefore, with the same vibration input and the addition of stochastic resonance, a greater number of mechanoreceptors can be activated, in a similar temporal pattern, resulting in a greater amount of sensory information being transmitted to spinal interneurons. These interneurons may release GABA or glycine (inhibitory neurotransmitters) onto the alpha motor neurons of tibialis anterior, and glutamate (an excitatory neurotransmitter) onto soleus alpha motor neurons (Li et al., 2003; Nishimaru and Kakizaki, 2009; Abraira and Ginty, 2013; Bui et al., 2013). With an increased contribution from FAI afferents, there would be a recruitment of more and larger motor units in the soleus due to increased excitatory input onto this motorneuron pool. For tibialis anterior, there would be an increase in inhibitory input, resulting in fewer activated motor units, and therefore a smaller response. While it has not been supported, there is some suggestion that the middle-latency response may be transcortical (Gill, 2016). If this were the case, it is feasible that tactile noise may have enhanced corticomuscular coherence, as has been shown previously (Trenado et al., 2014a).

### Early and Late Responses

In addition to responses observed within cutaneous reflex latencies, significant responses were also observed at earlier (~30 ms) and later (>110 ms) latencies. The observed earlier latency response may arise from muscle Ia afferents embedded within the SOL and TA muscles. The vibration intensity applied to generate cutaneous responses was set to 10× the perceptual threshold. While not painful, this stimulus is quite intense and may have resulted in the vibration of tendons of these muscles causing the activation of Ia afferents. Interestingly, this early response appears to be modulated by tactile noise similar to the 70–110 ms response. This may be occurring due to enhanced cutaneous modulation of the fusimotor system. Previous work has shown that the activation of cutaneous afferents can modulate the firing of spindles (Aniss et al., 1990; Gandevia et al., 1994), likely through reflexive changes in gamma motor neuron excitability (Hunt, 1951; Hunt and Paintal, 1958; Gandevia et al., 1994). Therefore, it may be possible that when 20% PT noise was applied, enhanced cutaneous afferent information may have increased SOL spindle sensitivity and reduced TA spindle sensitivity. Alternatively, cutaneous activation does evoke early latency responses (Burke et al., 1991; Brooke et al., 1999; Zehr et al., 2001a), and thus, there is a possibility that the observed early latency responses may be of cutaneous origin. However, these responses typically occur with latencies of ~50 ms. Therefore, we do not believe that the responses occurring at 30 ms in our data are of cutaneous origin. While the origin of the responses observed at the later latencies is unknown, they may arise from transcortical cutaneous pathways which have been shown in our previous work (Gill, 2016).

### Implications for Interventions

Healthy aging involves morphological and physiological changes to cutaneous mechanoreceptors that impair their ability to perform sensory functions and to reflexively modulate muscle activity in the lower limb (Peters et al., 2016). These impairments may contribute to increased fall risks in this population. Our work suggests that the addition of tactile noise, particularly at an intensity around 20% PT is a viable method to enhance cutaneous reflex responses in the lower limb, particularly in the musculature that facilities control at the ankle. These findings may inform the design of clinical interventions such as shoe insoles that incorporate tactile noise components to enhance the cutaneous reflex generation and consequently improve balance performance. Further work exploring the effect of subthreshold tactile noise on cutaneous reflex generation in such populations is required. Additionally, future work should aim to quantify SR-related enhancements across other mechanoreceptor classes using more specific noise stimuli and whether there is any evidence that enhancements are sustained after the noise stimulus is removed.

## Conclusions

Our work provides evidence that the addition of tactile electrical noise can enhance the generation of cutaneous reflex responses, specifically when targeting FAI receptors. When applied to the heel, the optimal intensity of noise is approximately 20% PT. This suggests that subthreshold tactile noise stimuli can be used to improve the generation of reflex responses from the cutaneous mechanoreceptors of the plantar foot. With further research, it is possible to incorporate such noise components into devices such as insoles to improve reflex generation, with the hope of improving postural balance and gait.

## Data Availability Statement

The raw data supporting the conclusions of this article will be made available by the authors, without undue reservation.

## Ethics Statement

The studies involving human participants were reviewed and approved by University of Guelph Research Ethics Board. The patients/participants provided their written informed consent to participate in this study.

## Author Contributions

TS, RP, and LB contributed substantially to the conception and design of the study. TS collected the data and all authors were substantially involved in the analysis and interpretation of the data. TS wrote the initial draft of the manuscript. TS, RP, and LB revised this draft before submission.

## Conflict of Interest

The authors declare that the research was conducted in the absence of any commercial or financial relationships that could be construed as a potential conflict of interest.
